# Ultra-high performance liquid chromatography high-resolution mass spectrometry for metabolomic analysis of dental calculus from Duke Alessandro Farnese and Maria D’Aviz

**DOI:** 10.1038/s41598-023-36177-2

**Published:** 2023-06-02

**Authors:** Nicolo’ Riboni, Federica Bianchi, Monica Mattarozzi, Marianna Peracchia, Marco Meleti, Maria Careri

**Affiliations:** 1 Department of Chemistry, Life Sciences and Environmental Sustainability, University of Parma, Parco Area Delle Scienze 17/A, 43124 Parma, Italy; 2 Department of Medicine and Surgery, Centro Universitario di Odontoiatria, University of Parma, Via Gramsci 14, 43126 Parma, Italy

**Keywords:** Metabolomics, Mass spectrometry, Mass spectrometry

## Abstract

Dental calculus is a valuable resource for the reconstruction of dietary habits and oral microbiome of past populations. In 2020 the remains of Duke Alessandro Farnese and his wife Maria D’Aviz were exhumed to get novel insights into the causes of death. This study aimed to investigate the dental calculus metabolome of the noble couple by untargeted metabolomics. The pulverized samples were decalcified in a water-formic acid mixture, extracted using methanol/acetonitrile and analyzed by ultra-high performance liquid chromatography coupled to high-resolution mass spectrometry (UHPLC-HRMS) using a reversed-phase separation followed by electrospray ionization and full scan in positive and negative ion mode. Waters Synapt-G2-Si High-Definition hybrid quadrupole time-of-flight mass spectrometer was used. Significant features were then identified using MS^E^ acquisition mode, recording information on exact mass precursor and fragment ions within the same run. This approach, together with data pre-treatment and multivariate statistical analysis allowed for the identification of compounds able to differentiate between the investigated samples. More than 200 metabolites were identified, being fatty acids, alcohols, aldehydes, phosphatidylcholines, phosphatidylglycerols, ceramides and phosphatidylserines the most abundant classes. Metabolites deriving from food, bacteria and fungi were also determined, providing information on the habits and oral health status of the couple.

## Introduction

Dental calculus is a mineralized microbial plaque, which accumulates at the surface of the tooth^1^. The mineral is deposited from crevicular fluid, but ultimately derives by precipitation of salivary calcium salts and for this reason the concentration of calculus is greater in the sites closest to the ducts of the salivary glands^2^. Being mainly composed of inorganic constituents, among which hydroxyapatite, fluorapatite, octacalcium phosphate and whitlockite^3^, dental calculus is well-preserved in archaeological samples and it can entomb biomolecules (e.g., DNA, proteins and lipids) associated to the oral microbiota, the host, and microdebris of exogenous origin^4–7^. Therefore, in the past two decades dental calculus has been an important resource to investigate health status, lifestyle and diet in the past populations. Optical microscopy^8^, scanning electron microscopy, also coupled to energy dispersive X-ray spectroscopy^9,10^, pyrolysis–gas chromatography-mass spectrometry^11^ and multi-omics techniques including proteomics, genomics and metabolomics^12,13^ proved to be valuable techniques to characterize exogenous debris entrapped in dental calculus, providing information on habits and health of individuals living in the past. Being able to provide a comprehensive fingerprinting of the investigated samples, mass spectrometry-based omics strategies are particularly promising thanks to their sensitivity, high-throughput and discriminating power. One of the most important advantages of these techniques relies on their untargeted nature, thus providing information on a plethora of biomolecules and allowing biomarker identification.

Combined to the use of bioinformatics and computational approaches, metabolomics plays a key role in understanding metabolome and in the identification of metabolites present in biological samples^14–16^. Different analytical platforms, among which high resolution mass spectrometry (HRMS) coupled to both gas- and liquid chromatography (GC-HRMS, GCxGC-HRMS and LC-HRMS) and nuclear magnetic resonance (NMR) can be applied to achieve a comprehensive covering of metabolome. Due to the enhanced specificity, sensitivity and availability of large spectral databases compared to NMR, HRMS has been widely applied for the metabolomics profiling of different biofluids, tissues and other biological samples^17–19^. In particular, analytical strategies involving on-line coupling of orthogonal UHPLC separations with HRMS represent the best tools to extend metabolite coverage^20^, because UHPLC ranks among the most efficient separation techniques^21^ and its combination with HRMS allows for the identification of a broad range of metabolites^22^.

Recently, Velsko and coauthors investigated the dental calculus metabolome in both modern and ancient specimens by using targeted and untargeted MS-based approaches^23^. In particular, the use of both UHPLC coupled with high-resolution tandem mass spectrometry (UHPLC-HRMS/MS) and GC-HRMS allowed the identification of metabolites belonging to different chemical classes, mainly lipids and amino acids, together with several xenobiotic compounds, providing insight into microbiome activity, metabolic processes, and degradation patterns.

In the present study, UHPLC-HRMS using a Waters hybrid quadrupole time-of-flight Synapt-G2-Si High-Definition (HD)MS QTOF mass spectrometer was applied to investigate the metabolome of dental calculus from Duke Alessandro Farnese and his wife Maria D’Aviz, who ruled the Duchy of Parma and Piacenza in the sixteenth century. Alessandro Farnese (1545–1592), a key member of the court of Philip II of Spain, was among the most influential military and political leaders of the sixteenth century and pursued numerous victories, particularly in Flanders, where he was governor for 15 years. The envy aroused by his success led to suspicion that his death could be caused by poisoning. To dispel this doubt, in 2020 the remains of both Alessandro Farnese and his wife were exhumed from the crypt of the Basilica of Santa Maria della Steccata in Parma (Italy). After opening the lead case containing the remains, the teeth were submitted to morphological analysis. The main feature in the dentition of Alessandro Farnese was severe wear, whereas the teeth of Maria D’Aviz were characterized by the presence of caries and periodontal diseases^24^. These findings could be associated with the different lifestyle of the noble couple, Duke Farnese spending most of his adult lifetime on battlefields all over Europe, and his wife maintaining the habits of the Portuguese court in the city of Parma. In this context, we tested the capability of UHPLC-HRMS operating in scanning MS^E^ mode to investigate the differences in the metabolomics profile of dental calculus of the duke and his wife, with the final aim of achieving novel insights on Alessandro Farnese’s causes of death, as well on the habits, health state and diet of the noble couple.

## Results and discussion

HRMS analysis was applied to achieve a highly confident identification of the metabolites present in the dental calculus of the noble couple. The capabilities of the Waters Synapt G2-Si HD mass spectrometer for complex matrix profiling were exploited by operating in MS^E^ mode: the use of an alternate scanning acquisition of both low and high-energy profiles provides information on both precursor and fragment ions in a single run with superior duty cycle to other data independent analysis techniques.

**Figure 1:**
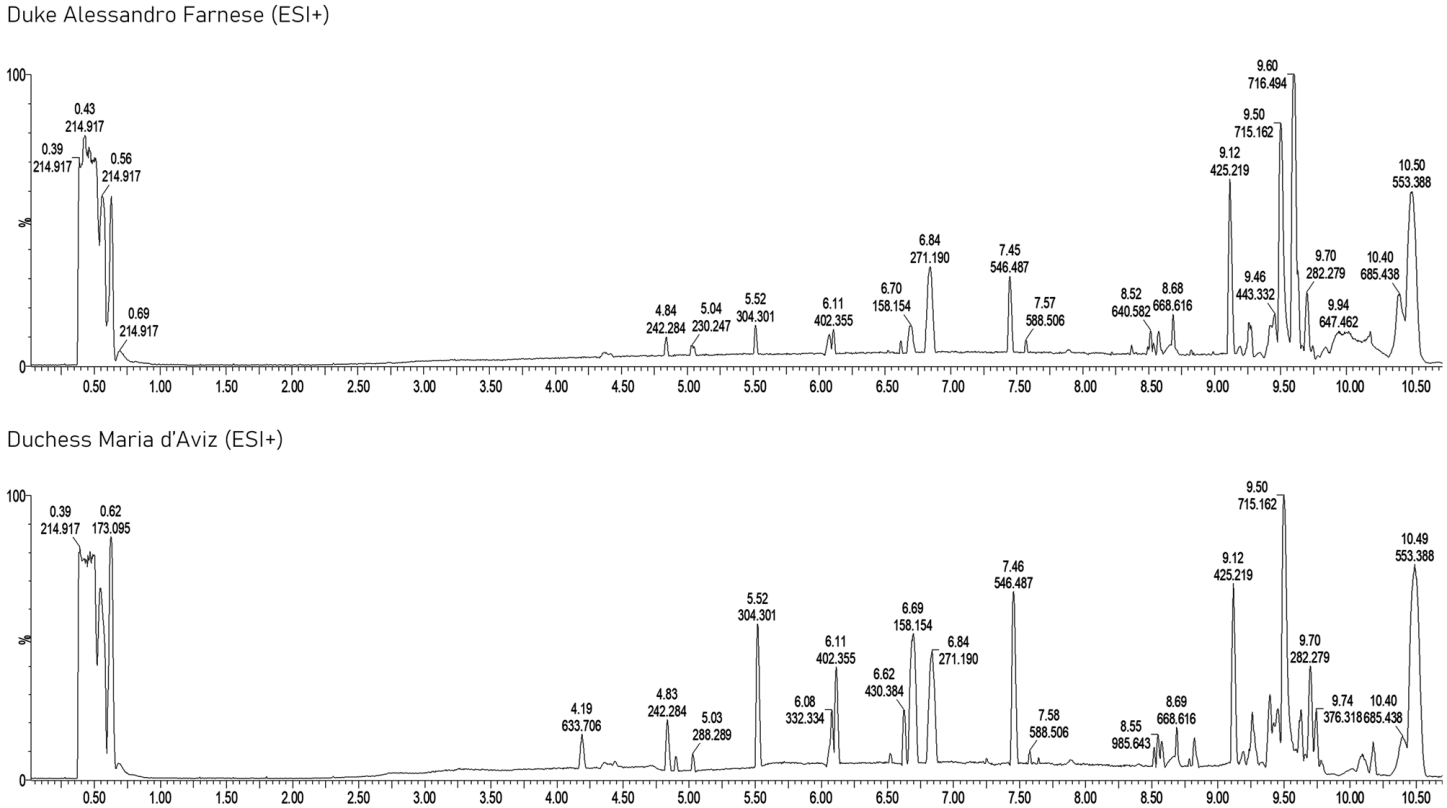
UHPLC-HRMS chromatograms in PI mode of dental calculus samples from: top) Maria D’Aviz and bottom) Duke Alessandro Farnese. Column: Atlantis Premier BEH C18 AX 1.7 μm (2.1 × 100 mm); mobile phase: (**A**) water + 0.1% (*v/v*) formic acid and (**B**) acetonitrile + 0.1% (*v/v*) formic acid; injection volume: 8 µL.

Due to the low amount of sample available, the most suitable experimental conditions for both extraction and analysis were preliminary assessed using modern dental calculus samples by operating both in the PI and NI mode. The effects of both the ratio between the aqueous and organic phase for the extraction procedure and the injection volume were investigated: a 1:3 ratio was used to induce protein precipitation, avoiding a high dilution of the extracted metabolites, while 8 µL were injected to obtain adequate signal intensities to perform measurements (Fig. 1). A good repeatability was also obtained in terms of area of selected chromatographic peaks with relative standard deviations always lower than 5 and 7% for PI and NI modes, respectively.

### Metabolomic analysis

The high number of features generated by HRMS is one of the most challenging aspects of untargeted metabolomics: in this context, both filtering and data reduction strategies need to be applied to select the significant *m/z* values for their subsequent identification based on the measured accurate mass of both precursor and fragment ions.

A total of 5918 and 5689 features were extracted in the dental calculus sample of Duke Alessandro Farnese by operating in PI and NI mode, respectively, whereas 5525 and 4728 features were recorded in the specimen of his wife Maria D’Aviz. To highlight the features responsible for the differentiation between the samples of the noble couple, only those having an intra-group variability of maximum 10% were used for subsequent processing. A minimum fold change of 3 compared to the extraction blank samples was also applied to correct the level of noise in the chromatograms of dental calculus samples. Finally, a value of 0.8 was set as reference value for statistical power analysis to detect real effects in the analyzed dataset. By applying the above-mentioned filters, a total of 4859 features was obtained.

**Figure 2:**
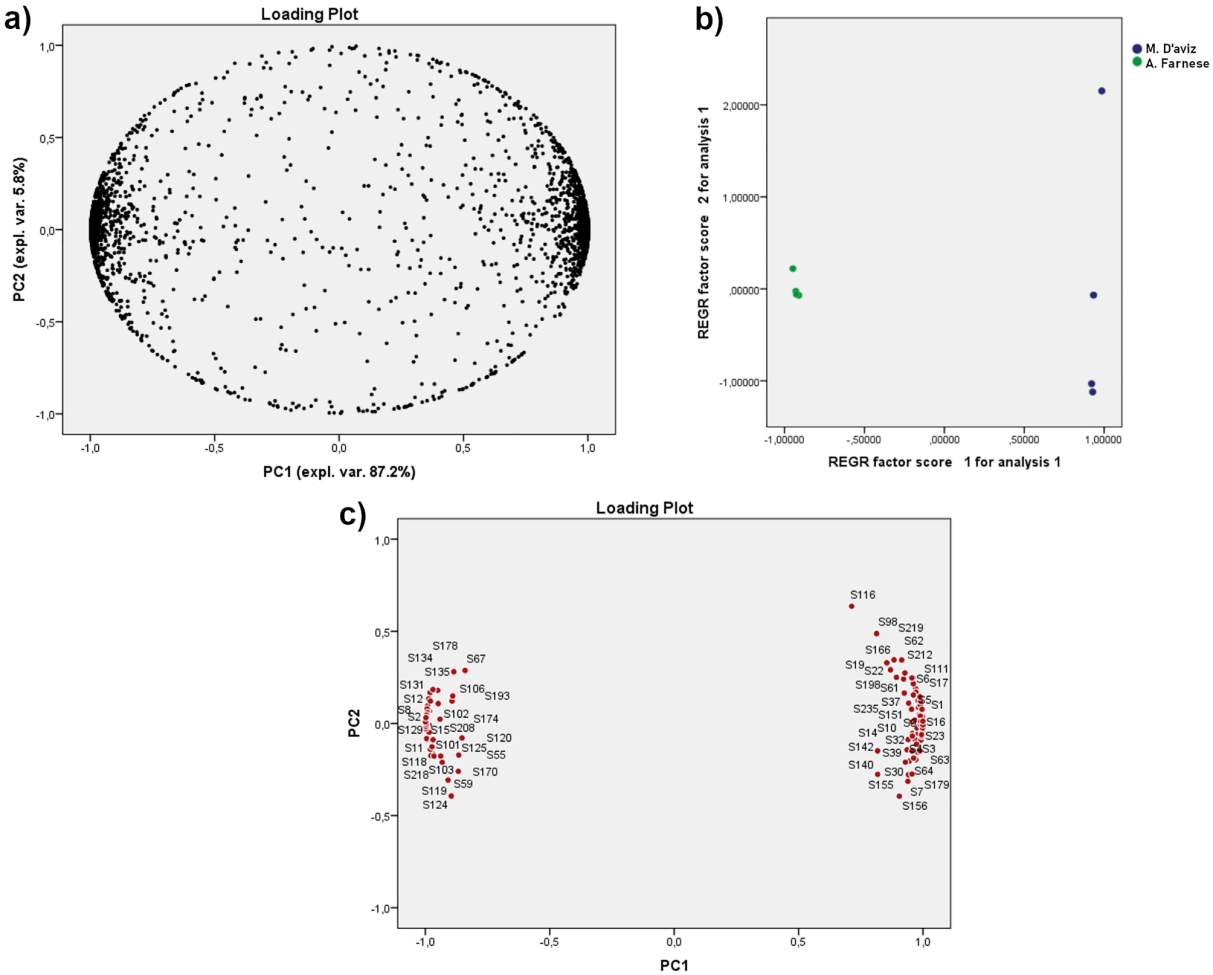
PCA: (**a**) loading plot and (**b**) score plot of all features; (**c**) highlight of the features having |loading |> 0.8.

Multivariate statistical analysis was performed using principal component analysis (PCA) with 99% of the total variance explained by the first 6 PCs. As shown in Fig. 2, a good separation between the dental calculus samples of Duke Alessandro Farnese and his wife was achieved along PC1, accounting for more than 87% of the total variance. A further reduction in the number of features (4227) was achieved by submitting to the identification process only the variables having an absolute score in the loading plot higher than 0.8 on PC1 (Fig. 2c).

Taking into account that no a priori information was available for elucidation purposes, compounds identification was performed considering all the information deriving from accurate mass measurements, fragmentation studies, analysis of the isotopic pattern, library matching and score fit.

A total of 217 metabolites belonging to different classes were identified (Table S1). Sphingolipids, glycerophospholipids and fatty acyls were the most abundant chemical classes, accounting for 132 compounds. The presence of a high number of lipids is not surprising: in a previous study on modern and ancient metabolome of dental calculus, Velsko et al. highlighted the influence of aqueous solubility on the preservation of compounds, assuming that compounds characterized by high water solubility could be degraded more easily^23^. Based on these findings, the non-polar nature of lipids can favor their preservation over time. Furthermore, the presence of saturated fatty acids was almost double than of unsaturated ones: according to Rogoz et al.^12^, this behavior can be ascribed to the reduced oxidative stability of unsaturated compounds, thus leading to an easier degradation. Eleven amino acids were detected: these compounds could also undergo modification over time; for example, the presence of tyramine can be related to the decarboxylation of tyrosine as a result of fermentation or bacterial decomposition processes. Tyramine was mostly detected in the dental calculus of Duke Alessandro Farnese. Considering that the duke spent most of his lifetime on the battlefields in Northern Europe, the presence of tyramine could be related to the consumption of long-life foods, such as seasoned cheese, dried or smoked herring, venison, cured meats and fermented beverages^25^.

Among the compounds potentially ascribable to the Duke’s eating habits, cucurbitin and catechin 5-*O*-beta-d-glucopyranoside-4'-Me could be related to the consumption of typical foods present on the tables of Northern Europe such as Cucurbitaceae and rhubarb^26,27^. By contrast, 3-hydroxy-3-methyloxindole, imidazoleacetic acid riboside, 2-(methylamino)-1-phenylethanol and arginyl hydroxyproline can be related to the eating habits of Maria D’Aviz: in fact, according to Human Metabolome DataBase and Food DataBase (FooDB), these compounds could be considered potential biomarkers for the consumption of anatidaes, chickens, and domestic pigs and it is known that the Maria D’Aviz used poultry as main ingredient of several recipes described in her famous cookbook^28^.

Among metabolites commonly found in spices and tea, salicylaldehyde, DAG(32:4), capsiamide, estragole, phenylpyridines and eugenol quinone methide (EQM) were some of the compounds detected mostly in the dental calculus of Maria D’Aviz. The consumption of these foods is not surprising: Maria D’Aviz was a member of the Portuguese court, and the spices, which are frequently cited in the cookbook of the noblewoman, could easily be imported from both the Azores, the most important colony of the Portuguese empire and Asia^29^.

As for salicylates, they are contained in herbs, spices and tea^30^ and it is known that extracts of plants rich in salicylates, owing to their anti-inflammatory and anti-pyretic effects, have been used in ancient time to treat different human diseases^31^.

The presence of EQM, an oxidation derivative of eugenol^32,33^ can be explained not only by considering the dietary intake of spices such as cloves, nutmeg and cinnamon^34^, but also the anti-inflammatory and analgesic effects of eugenol-based remedies^35^, which have been used to treat toothache, a pathology that has affected Maria D’Aviz. However, the presence of EQM could also be ascribed to the use of essences for embalming, a practice used by the nobles in the sixteenth century^36,37^. Similarly, the presence of other metabolites deriving from fruits and citrus like hesperidin, neohesperidin, narirutin, naringin chalcone, dihydroxycitracridone I, amphibine H, which were observed in the dental calculus of the noble couple, could be explained taking into account both food consumption and their use as perfumes and essences used in the embalming practice.

With regard to the oral microbiome, some of the compounds detected can also be ascribed to the metabolism of fungi and bacteria. An interesting result is related to the presence of the TMC-34 metabolite deriving from *Actinomyces,* which are involved in the development of periodontitis^38–40^. This metabolite was mainly present in the dental calculus of Maria D’Aviz, and it is known that the noble woman suffered from caries and periodontal diseases^24^.

Additional metabolites like the glycerophospholipids PIM1(37:3), LPIM4(18:2) and LPIM4(19:2) related to the bacterium *Mycobacterium tuberculosis* were also identified^41^. After his death, the autopsy of Duke Alessandro Farnese revealed the presence of lung diseases^42^ further confirmed by the results achieved during exhumation in 2020^43^, thus suggesting pneumonia, a widespread disease in the fifteenth and sixteenth centuries^44,45^.

It has also to be highlighted the presence of different metabolites of *Saccharomyces cerevisiae,* namely C16 phytosphingosine, C20 phytosphingosine, MIPC 42:0;O3, MIPC 40:0;O2, PI-Cer(d46:0)^46^, which can be related to bread baking and fermentation processes associated with winemaking and brewing^47,48^. Finally, the presence of other metabolites like penipacid B (2-[[N-(2-methoxy-2-methylpropyl)-C-methylcarbonimidoyl]amino]benzoic acid), versixanthone E, asperversin G or 2,4,6,8-tetramethyl-3,4-dihydroxydec-8(9)-enolide, deriving from the *fungi Penicillium paneum*, *Aspergillus versicolor* and *Botrytis cinerea,* can be related to the eating habits of the noble couple. Being able to contaminate cereals, oilseeds and nuts, these molds are mainly involved in the spoilage of bread^49–51^.These findings suggest that also the members of upper classes living in the Renaissance consumed dried and poorly preserved bread.

## Conclusions

Untargeted metabolomics using UHPLC coupled to Waters Synapt G2-Si HDMS system was successfully applied to analyze the metabolome of the dental calculus of people living in ancient time. Concerning the untargeted metabolomics of Duke Alessandro Farnese and his wife Maria D’Aviz, the ability to efficiently separate the components of such complex samples combined with data independent acquisition by high resolution MS^E^ permits to maximize the information obtained from the samples investigated by recording both low-energy and high-collision energy profiles related to all compounds in a single run. UHPLC-HRMS followed by Principal Component Analysis was successfully used for differentiation of the samples of the noble couple allowing the identification of more than 200 metabolites able to provide novel insights on the dietary habits and health status of Duke Alessandro Farnese and his wife. From the result of metabolomics analysis, it suggested that combination of LC-HRMS with PCA offers a powerful analytical technique to differentiate dental calculus in ancient specimens and identify metabolites markers, which have essential roles in samples differentiation, According to the forensics analysis of the remains, the presence of metabolites related to *Mycobacterium tuberculosis* can be considered a valuable step to clarify the causes of the duke’s death, while the identification of compounds deriving from the consumption of food confirmed the different lifestyles of the noble couple.

## Materials and methods

### Chemicals and materials

LC–MS grade water, acetonitrile, methanol and formic acid were purchased from Honeywell Burdick & Jackson (Charlotte, NC, USA). Leucine enkephalin standard was obtained from the Waters TOF G2-S Sample Kit-1 (Waters, Milford, MA, USA).

### Dental calculus collection and metabolite extraction

Historic dental calculus was collected from the remains of Duke Alessandro Farnese and Maria D’Aviz after their exhumation in 2020 from the Basilica of Santa Maria della Steccata (Parma, Italy). Notary Dr. Marco Micheli in Parma granted permission for the exhumation and for the subsequent analysis. During procedures of physical examination of teeth, the couple’s calculus was collected with the use of a sterile curette following the protocols to avoid cross-contamination, then it was stored in glass vials until analysis^52,53^. According to the guidelines for the identification of cases to be submitted to the Ethical Committee of the Area Vasta Emilia Nord (https://www.aou.mo.it/ComitatoEticoAVEN), no approval was required.

Modern dental calculus samples from four healthy donors to be used for assessing both the extraction and chromatographic conditions were obtained during professional oral hygiene sessions. According to the guidelines for the identification of cases to be submitted to the Ethical Committee of the Area Vasta Emilia Nord (https://www.aou.mo.it/ComitatoEticoAVEN), no approval was required. Sample collection was performed in accordance with the Declaration of Helsinki. Informed consent was obtained from the donors.

After collection, both historical and modern dental calculus samples were frozen at -80 °C until metabolite extraction. Sample decalcification was performed according to Velsko et al.^23^ with slight modification: the samples (20 mg) were gently powdered in an agate mortar, placed in sterile glass vials and decalcified with 100 μL of 4% (*v/v*) formic acid in water at 4 °C for 18 days performing sonication at regular intervals. Samples to be submitted to UHPLC-HRMS analysis in ESI^−^ mode were neutralized by the addition of 15 µL of a 5 M ammonium hydroxide solution. Finally, 300 µL of an acetonitrile/methanol solution 1:1 (*v:v*) were added to the samples, which were centrifuged at 12,000×*g* at 4 °C for 30 min. The supernatant was then collected and submitted to the UHPLC-HRMS analysis. A sample obtained by mixing the extracts of both Duke Alessandro Farnese and his wife was used as quality control sample.

### Ultra-high performance liquid chromatography quadrupole high-resolution mass spectrometry

Chromatographic separation was performed on a binary Acquity UHPLC I-Class system (Waters) using an Atlantis™ Premier BEH™ C_18_ AX 1.7 μm (2.1 × 100 mm) column (Waters), thermostated at 40 °C. Mobile phase consisted of water (solvent A) and acetonitrile (solvent B), both containing 0.1% (*v/v*) formic acid. The flow rate was 0.4 mL/min and injection volume was 8 μL. A multistep linear gradient elution was carried out under the following conditions: solvent B was set at 2% for 1 min, followed by a linear gradient to 60% within 6 min, then to 95% in 1.5 min, maintained for 1.5 min before column re-equilibration (5 min). Elution was performed within 10 min.

HRMS analyses were performed on a Synapt G2-Si HDMS QTOF mass spectrometer (Waters SpA, Milan, Italy) with an electrospray ionization (ESI) Zspray™ (Waters) both in positive (PI) and negative (NI) ion modes. Mass correction during chromatographic runs was performed using leucine-enkephalin solution (50 ng/mL in acetonitrile/water, 50:50 (*v/v)* with 0.1% formic acid) as lock mass, by infusing it through the LockSpray™ system (Waters) with a 15 s interval. The experiments were conducted in resolution mode (20000 FWHM). Operating parameters were as follows: capillary voltage, 0.80 and 0.50 kV in ESI^+^ and ESI^−^ respectively; cone voltage, 50 V; source temperature, 150 °C; source offset 80 V; desolvation temperature, 600 °C; cone gas, 50 L/h; desolvation gas, 1000 L/h; nebulizer pressure 6.5 bar. Spectra were acquired operating in data independent MS^E^ acquisition mode by applying 5 V as collision energy for the low energy profile and using a collision energy ramp from 25 to 45 V for the high energy profile.

The UHPLC-HRMS data were recorded in *.raw files by using the MassLynx (v4.2) software (Waters).

### Data analysis

Data analysis was performed by processing the .raw data in Progenesis QI software (Waters, Milford, MA, USA). The software allows for data visualization analysis, creation of 2D maps and the analysis of chromatograms and spectra. The program provides auto-alignment of signals, peak peaking, deconvolution and normalization^54,55^. The following adducts were considered: [M+H]^+^, [M+Na]^+^, [M+K]^+^, [M+NH_4_]^+^, [M+H_2_O+H]^+^, [M−H_2_O+H]^+^, [M+2H]^2+^, [M+2Na]^2+^, [M+2Na−H]^+^, [2M+H]^+^, [2M+Na]^+^, [M+H+Na]^2+^ in PI, and [M−H]^−^, [M+H_2_O−H]^−^, [M−H_2_O−H]^−^, [M+HCOO]^−^, [2M−H]^−^, [M+Na−2H]^−^, [M+K−2H]^−^ in NI.

The data were filtered by setting a maximum intra-group variability of 10%, a power analysis value > 0.8 and a minimum fold change of 3 compared to method blank.

PCA was performed to explore the data set and to obtain the features able to differentiate samples belonging to Duke Alessandro Farnese and Maria D’Aviz. Compounds identification was performed by comparing the spectra with those stored in different ChemSpider online libraries, namely the HMDB, the FooDB, the *E. coli* Metabolome Database, the Yeast Metabolome Database, LipidMAPS, NPAtlas e KEGG Database, using a mass error tolerance of 5 ppm for precursor ions and 10 ppm for fragment ions.

### Lipid nomenclature

The LIPID MAPS® glycerophospholipid abbreviations (PC, PE, etc.) are used here to refer to the identified analytes.

## Supplementary Information

Supplementary Information.

## Data Availability

The datasets used and analyzed during the current study are available from the corresponding author upon reasonable request.
