# Supplementary material for: Ultra-high performance liquid chromatography high-resolution mass spectrometry for metabolomic analysis of dental calculus from Duke Alessandro Farnese and Maria D’Aviz
Source: Sci Rep. 2023 Jun 2;13:8967. doi: 10.1038/s41598-023-36177-2 (PMC10238497; doi:10.1038/s41598-023-36177-2)
Supplement: Supplementary file 1 — Supplementary Information. [file 41598_2023_36177_MOESM1_ESM.docx]

**Ultra-high performance liquid chromatography high-resolution mass spectrometry for metabolomic analysis of dental calculus from Duke Alessandro Farnese and Maria D’Aviz**

Nicolo’ Riboni^1^, Federica Bianchi^1*^, Monica Mattarozzi^1^, Marianna Peracchia^2^, Marco Meleti^2^, and Maria Careri^1^

^1^University of Parma, Department of Chemistry, Life Sciences and Environmental Sustainability, Parco Area delle Scienze 17/A, 43124 Parma, Italy

^2^University of Parma, Centro Universitario di Odontoiatria, Department of Medicine and Surgery, via Gramsci 14, 43126 Parma, Italy

Corresponding Author

*Federica Bianchi: e-mail: federica.bianchi@unipr.it

Table S1. Metabolites identified in the dental calculus samples

| **Compound** | **class** | **ESI Polarity** | **m/z** | **Detected Adduct** | **Error (ppm)** | **Retention time (min)** | **Anova (p)** | **q Value** | **Highest Mean** | **CSID** | **HMDB** |
| --- | --- | --- | --- | --- | --- | --- | --- | --- | --- | --- | --- |
| gamma aminobutyric acid | Amino acids | + | 86,060549 | [M-H_2_O+H]^+^ | 4,90 | 0,633 | 9,77E-05 | 8,28E-05 | Duchess | 116 | 0000112 |
| oxohexanoic acid | Fatty Acyls | + | 113,060213 | [M-H_2_O+H]^+^ | 3,95 | 8,034 | 7,76E-13 | 1,48E-11 | Duke | 17383 | 0061881 |
| 8-aminooctanoic acid | Fatty Acyls | + | 142,123249 | [M-H_2_O+H]^+^ | 3,81 | 9,702 | 2,80E-05 | 2,71E-05 | Duchess | 59474 |  |
| 10-aminodecanoic acid | Fatty Acyls | + | 170,154747 | [M-H_2_O+H]^+^ | 4,32 | 9,698 | 5,20E-05 | 4,71E-05 | Duchess | 75018 |  |
| dodecadienoic acid | Fatty Acyls | + | 179,142496 | [M-H_2_O+H]^+^ | -2,79 | 7,506 | 8,11E-05 | 7,00E-05 | Duchess | 4471815 |  |
| tridecanoic acid | Fatty Acyls | + | 197,189659 | [M-H_2_O+H]^+^ | -1,57 | 8,248 | 1,07E-04 | 8,99E-05 | Duchess | 12013 | 0000910 |
| hydroxytridecanoic acid | Fatty Acyls | - | 251,163137 | [M+Na-2H]^-^ | 1,16 | 10,051 | 6,61E-03 | 2,29E-02 | Duchess | 122656 |  |
| hydroxypentadecanoic acid | Fatty Acyls | - | 257,211009 | [M-H]^-^ | -4,63 | 9,681 | 4,31E-08 | 6,01E-07 | Duke | 70730 |  |
| hydroxyhexadecanoic acid | Fatty Acyls | + | 255,232359 | [M-H_2_O+H]^+^ | 1,87 | 5,573 | 6,78E-06 | 7,87E-06 | Duchess | 57263229 |  |
| octadecatetraenoic acid | Fatty Acyls | + | 259,205949 | [M-H_2_O+H]^+^ | 1,14 | 9,363 | 5,43E-06 | 6,46E-06 | Duchess | 4471932 |  |
| hydroxyoxohexadecanoic acid | Fatty Acyls | - | 285,206638 | [M-H]^-^ | -1,76 | 8,503 | 4,16E-04 | 1,93E-03 | Duke | 30777548 | 0041287 |
| hydroxymargaric acid | Fatty Acyls | - | 285,242983 | [M-H]^-^ | -1,86 | 10,613 | 2,07E-04 | 1,03E-03 | Duke | 3513977 |  |
| dehydrophytosphingosine | Sphingolipids | + | 316,221592 | [M+H]^+^ | -0,60 | 2,953 | 7,06E-04 | 4,91E-04 | Duke | 26333231 | 0038057 |
| docosadienoic acid | Fatty Acyls | + | 319,300036 | [M-H_2_O+H]^+^ | 1,46 | 8,785 | 4,07E-04 | 2,98E-04 | Duchess | 113368502 |  |
| pimelic acid | Fatty Acyls | - | 181,048862 | [M+Na-2H]^-^ | 3,95 | 3,284 | 3,64E-08 | 5,24E-07 | Duke | 376 | 0000857 |
| diethyl succinate | Fatty Acyls | - | 195,064703 | [M+Na-2H]^-^ | 4,78 | 3,483 | 4,71E-04 | 2,16E-03 | Duke | 13865630 | 0033838 |
| myristic acid | Fatty Acyls | - | 227,200666 | [M-H]^-^ | -4,30 | 9,726 | 9,84E-03 | 3,26E-02 | Duchess | 10539 | 0000806 |
| palmitic acid | Fatty Acyls | - | 255,232155 | [M-H]^-^ | -3,82 | 10,430 | 1,07E-04 | 5,60E-04 | Duchess | 960 | 0000220 |
| ethyl stearate | Fatty Acyls | + | 335,292828 | [M+Na]^+^ | 2,50 | 9,397 | 6,91E-09 | 2,20E-08 | Duchess | 7830 | 0034156 |
| 9-Octadecenamide | Fatty Acyls | + | 282,280319 | [M+H]^+^ , [M+Na]^+^ | 4,24 | 9,702 | 2,60E-07 | 4,73E-07 | Duchess | 4446508 | 0002117 |
| anandamide (20:2, n-6) | Fatty Acyls | + | 374,304165 | [M+H]^+^ , [M+Na]+ , [M+K]+ | 3,48 | 9,397 | 3,18E-09 | 1,20E-08 | Duchess | 4446564 |  |
| anandamide (20:1, n-9) | Fatty Acyls | + | 376,318534 | [M+Na]^+^ , [M-H_2_O+H]^+^ | -0,18 | 9,215 | 4,94E-07 | 8,24E-07 | Duchess | 4446572 |  |
| tricosanoyl ethanolamine | Fatty Acyls | + | 436,353893 | [M+K]^+^ | -3,10 | 6,769 | 4,61E-03 | 2,68E-03 | Duchess | 24842026 |  |
| nervonoyl ethanolamine | Fatty Acyls | + | 448,354383 | [M+K]^+^ | -1,82 | 7,744 | 1,05E-07 | 2,16E-07 | Duchess | 21169186 |  |
| N-(6-aminohexanoyl)-6-aminohexanoic acid | Fatty Acyls | - | 243,170527 | [M-H]^-^ | -3,66 | 2,559 | 1,81E-07 | 1,96E-06 | Duke | 871 |  |
| 7-epiloganic acid | Glycosides | + | 359,134403 | [M-H_2_O+H]^+^ | 2,02 | 3,581 | 5,18E-05 | 4,69E-05 | Duchess | 391578 |  |
| alpha-N-(3-hydroxyhexadecanoyl) L-ornithine | Fatty Acyls | + | 387,321938 | [M+H]^+^ , [M-H_2_O+H]^+^ | 0,54 | 5,941 | 8,76E-08 | 1,85E-07 | Duke | 113371144 |  |
| hydroxy(octanoyloxyl)propyl decanoate | Glycerolipids | + | 395,275975 | [M+Na]^+^ | -2,18 | 8,760 | 6,89E-03 | 3,86E-03 | Duchess | 34989566 | 0092959 |
| [hexadecenoyloxy]decanoic acid | Fatty Acyls | + | 407,351257 | [M+H]^+^ , [M-H_2_O+H]^+^ | -1,67 | 8,324 | 1,62E-05 | 1,67E-05 | Duke | 113368738 |  |
| pentacosenoic acid | Fatty Acyls | + | 425,336169 | [M+2Na-H]^+^ | 2,94 | 6,799 | 9,62E-06 | 1,06E-05 | Duke | 10476097 |  |
| dihydroxy-3-oxo-4-cholenoic acid | Sterol Lipids | + | 427,245976 | [M+Na]+ , [M+K]+ | 1,16 | 9,388 | 6,74E-03 | 3,79E-03 | Duchess | 4447084 | 0000447 |
| Tetracosyl-hexadecenoate | Fatty Acyls | + | 635,571210 | [M+2Na-H]^+^ | -0,19 | 7,369 | 0,00E+00 | 0,00E+00 | Duchess | 113368523 |  |
| trihydroxyoctadecanoic acid | Fatty Acyls | - | 663,502626 | [2M-H]^-^ | -4,02 | 10,621 | 8,82E-04 | 3,81E-03 | Duchess | 24022689 | 0030935 |
| cis-hept-4-enol | Fatty Acyls | + | 97,101464 | [M-H_2_O+H]^+^ | 2,55 | 9,702 | 2,92E-05 | 2,81E-05 | Duchess | 4519030 | 0031405 |
| phenylethyl alcohol | Primary alcohol | + | 105,070081 | [M-H_2_O+H]^+^ | 1,66 | 6,596 | 8,30E-04 | 5,67E-04 | Duchess | 5830 | 0033944 |
| octenol | Fatty Acyls | + | 111,117044 | [M-H_2_O+H]^+^ | 1,68 | 9,702 | 1,07E-05 | 1,16E-05 | Duchess | 4517064 | 0031296 |
| octatrienal | Fatty Acyls | + | 105,070309 | [M-H_2_O+H]^+^ | 3,53 | 8,591 | 1,51E-03 | 9,73E-04 | Duchess | 4446452 |  |
| 2-butylfuran | Heteroaromatic compounds | + | 107,085952 | [M-H_2_O+H]^+^ | 3,44 | 9,788 | 5,32E-06 | 6,34E-06 | Duke | 19338 | 0040272 |
| nonadienal | Fatty Acyls | + | 121,101252 | [M+H]^+^ , [M-H_2_O+H]^+^ | 0,57 | 9,706 | 1,86E-04 | 1,47E-04 | Duchess | 4509638 | 0031152 |
| salicylaldehyde | Substituted aromatic compounds | + | 123,044663 | [M+H]^+^ | 4,96 | 7,449 | 3,56E-05 | 3,35E-05 | Duchess | 13863618 | 0034170 |
| estragole | Substituted aromatic compounds | + | 131,085067 | [M-H_2_O+H]^+^ | -3,15 | 8,684 | 1,08E-03 | 7,24E-04 | Duchess | 13850247 | 0034121 |
| dihydrojasmone | Substituted aromatic compounds | + | 149,132823 | [M-H_2_O+H]^+^ | 2,13 | 9,702 | 2,09E-05 | 2,09E-05 | Duchess | 56166 | 0031565 |
| tetradecadienal | Fatty Acyls | + | 231,172934 | [M+Na]^+^ | 4,83 | 7,287 | 9,27E-09 | 2,79E-08 | Duchess | 24841994 |  |
| octadecadienal | Fatty Acyls | + | 265,253598 | [M+H]^+^ , [M-H_2_O+H]^+^ | 3,75 | 9,702 | 3,50E-07 | 6,07E-07 | Duchess | 24842015 |  |
| sphingosine d14:1 | Sphingolipids | + | 226,217014 | [M-H_2_O+H]^+^ | 1,93 | 4,640 | 3,60E-11 | 3,06E-10 | Duchess | 24823204 |  |
| hexadecasphinganine | Sphingolipids | + | 256,264103 | [M-H_2_O+H]^+^ | 2,22 | 8,944 | 1,36E-03 | 8,88E-04 | Duchess | 571105 |  |
| 14-methylhexadecasphingosine | Sphingolipids | + | 268,263920 | [M-H_2_O+H]^+^ | 1,48 | 9,139 | 1,13E-09 | 5,19E-09 | Duke | 24823209 |  |
| 15-methylhexadecasphingosine | Sphingolipids | + | 268,263981 | [M-H_2_O+H]^+^ | 1,70 | 9,447 | 5,73E-04 | 4,07E-04 | Duchess | 24823208 |  |
| sphinganine(d-17:0) | Sphingolipids | + | 270,278407 | [M-H_2_O+H]^+^ | -2,59 | 5,122 | 1,34E-04 | 1,10E-04 | Duchess | 2497508 |  |
| C16 phytosphingosine | Sphingolipids | + | 272,258653 | [M-H_2_O+H]^+^ | 0,88 | 5,784 | 3,92E-08 | 9,37E-08 | Duchess | 9487275 |  |
| sphingoadenine | Sphingolipids | + | 280,263366 | [M-H_2_O+H]^+^ | -0,44 | 8,413 | 1,20E-11 | 1,27E-10 | Duke | 24823205 |  |
| c17 sphingosine | Sphingolipids | + | 286,273877 | [M+H]^+^ | -0,65 | 8,989 | 1,17E-09 | 5,29E-09 | Duke | 4446670 |  |
| spisulosine | Sphingolipids | + | 286,310422 | [M+H]^+^ | -0,06 | 5,550 | 3,53E-12 | 4,77E-11 | Duchess | 8101521 |  |
| sphinga-4E,8E-dienine | Sphingolipids | + | 280,264024 | [M-H_2_O+H]^+^ | 1,77 | 8,328 | 1,18E-06 | 1,71E-06 | Duke | 9395282 |  |
| D-erythro-Sphingosine | Sphingolipids | + | 300,290217 | [M+H]^+^ | 1,67 | 4,939 | 1,09E-06 | 1,60E-06 | Duke | 4444047 |  |
| sphinganine d18:0 | Sphingolipids | + | 302,305663 | [M+H]^+^ , [M-H_2_O+H]^+^ | 1,07 | 5,757 | 1,61E-09 | 6,83E-09 | Duke | 82609 | 0000269 |
| hydroxy-sphingenine | Sphingolipids | + | 316,284433 | [M+H]^+^ | 2,51 | 4,559 | 4,53E-05 | 4,16E-05 | Duke | 26333231 |  |
| C18 phytosphingosine | Sphingolipids | + | 318,300675 | [M-H_2_O+H]^+^ | 1,60 | 5,485 | 6,69E-08 | 1,46E-07 | Duke | 108921 | 0004610 |
| C20 phytosphingosine | Sphingolipids | + | 328,320666 | [M-H_2_O+H]^+^ | -0,94 | 9,912 | 2,14E-06 | 2,85E-06 | Duke | 28184721 |  |
| sphinganine c20 | Sphingolipids | + | 330,337053 | [M+H]^+^ , [M-H_2_O+H]^+^ | 1,07 | 6,278 | 1,67E-09 | 6,99E-09 | Duke | 23253704 |  |
| ketosphinganine | Sphingolipids | + | 344,253138 | [M+2Na-H] ^+^ | -1,56 | 3,014 | 2,15E-08 | 5,63E-08 | Duke | 388895 | 0001480 |
| glycerophosphorylethanolamine | Glycerophospholipids | + | 198,052086 | [M-H_2_O+H]^+^ | -2,34 | 6,017 | 2,00E-06 | 2,70E-06 | Duchess | 4573608 | 0059660 |
| PG(15:0) | Glycerophospholipids | + | 493,253310 | [M+Na]^+^ , [M+K]^+^ | -0,80 | 8,034 | 3,95E-10 | 2,16E-09 | Duke | 113377837 |  |
| PG(26:0) | Glycerophospholipids | + | 677,376764 | [M+K]^+^ | -3,58 | 7,191 | 7,98E-03 | 4,43E-03 | Duke | 74878391 | 0116679 |
| PG(33:0) | Glycerophospholipids | - | 757,475804 | [M+K-2H]^-^ | 4,56 | 9,294 | 5,70E-03 | 2,01E-02 | Duke | 113377753 |  |
| PG(33:1) | Glycerophospholipids | + | 773,469564 | [M+K]^+^ | -4,57 | 4,751 | 1,44E-04 | 1,17E-04 | Duke | 59695439 |  |
| PG(35:5) | Glycerophospholipids | + | 799,448774 | [M+2Na-H] ^+^ | -1,12 | 7,771 | 1,75E-06 | 2,40E-06 | Duke | 113376907 |  |
| PGP(24:0) | Glycerophospholipids | + | 713,340135 | [M+Na]^+^ | 0,03 | 7,437 | 4,51E-03 | 2,63E-03 | Duke | 74878248 | 0116536 |
| PG(38:7) | Glycerophospholipids | + | 837,462423 | [M+2Na-H] ^+^ | -3,64 | 7,590 | 1,37E-03 | 8,94E-04 | Duke | 24768098 | 0010599 |
| SQDG 32:1 | Glycerolipids | + | 775,500882 | [M-H_2_O+H]^+^ | -1,98 | 6,903 | 7,01E-04 | 4,87E-04 | Duchess | 113374818 |  |
| PG(37:7) | Glycerophospholipids | - | 815,424685 | [M+K-2H]^-^ | -3,05 | 8,354 | 1,24E-03 | 5,19E-03 | Duchess | 113377041 |  |
| CDP-DG(36:3) | Glycerophospholipids | + | 986,524669 | [M-H_2_O+H]^+^ | -2,00 | 7,237 | 1,01E-04 | 8,54E-05 | Duchess | 74877705 | 0115967 |
| CDP-DG(41:0) | Glycerophospholipids | + | 1080,659370 | [M+H]^+^ | -2,83 | 8,817 | 3,62E-05 | 3,41E-05 | Duchess | 74878006 | 0116293 |
| CDP-DG(35:0) | Glycerophospholipids | + | 498,785994 | [M+2H]^2+^ | -3,80 | 0,606 | 2,44E-06 | 3,20E-06 | Duke | 74877811 | 0116078 |
| CDP-DG(42:2) | Glycerophospholipids | + | 1090,650441 | [M+H]^+^ | 3,34 | 0,539 | 3,55E-06 | 4,45E-06 | Duchess | 74877800 | 0116063 |
| CDP-DG(42:2) | Glycerophospholipids | - | 1126,593388 | [M+K-2H]^-^ | 4,82 | 0,631 | 5,59E-03 | 1,98E-02 | Duke | 748878061 | 0116348 |
| MG(16:0) | Glycerolipids | + | 353,266962 | [M+Na]^+^ , [M-H_2_O+H]^+^ | 2,22 | 9,603 | 7,42E-03 | 4,14E-03 | Duke | 2341519 | 0011564 |
| MGDG 36:7 | Glycerolipids | + | 801,491006 | [M+2Na-H] ^+^ | 2,91 | 5,130 | 1,13E-09 | 5,19E-09 | Duke | 113374853 |  |
| MG(22:1) | Glycerolipids | - | 433,329139 | [M+Na-2H]- | -1,90 | 9,865 | 2,59E-05 | 1,54E-04 | Duchess | 24765800 | 0011582 |
| DG(32:4) | Glycerolipids | - | 605,440222 | [M+FA-H]- | -3,70 | 10,567 | 2,38E-04 | 1,16E-03 | Duchess | 24765853 | 0007019 |
| Cer 33:0;O2 | Sphingolipids | + | 526,518480 | [M+H]^+^ | -1,68 | 8,393 | 2,13E-05 | 2,12E-05 | Duke | 113381263 |  |
| Cer 31:0;O2 | Sphingolipids | + | 542,452810 | [M+2Na-H] ^+^ | 1,69 | 7,118 | 4,09E-08 | 9,71E-08 | Duchess | 113381262 |  |
| Cer 33:2;O2 | Sphingolipids | + | 544,472523 | [M+Na]^+^ | 4,85 | 7,291 | 7,31E-12 | 8,47E-11 | Duchess | 113381092 |  |
| Cer 31:0;O3 | Sphingolipids | + | 558,449210 | [M+2Na-H] ^+^ | 4,55 | 6,217 | 2,17E-07 | 4,07E-07 | Duchess | 78315792 |  |
| Cer 34:2;O2 | Sphingolipids | + | 558,487158 | [M+Na]^+^ | 2,76 | 7,384 | 6,28E-04 | 4,42E-04 | Duke | 113381093 |  |
| Cer 36:3;O3 | Sphingolipids | + | 560,503972 | [M-H_2_O+H]^+^ | 0,42 | 7,525 | 7,12E-13 | 1,38E-11 | Duke | 113381135 |  |
| Cer 33:1;O3 | Sphingolipids | + | 562,482446 | [M+Na]^+^ | 3,51 | 6,861 | 1,26E-06 | 1,81E-06 | Duchess | 113381202 |  |
| Cer 34:1;O4 | Sphingolipids | + | 570,507139 | [M+H]^+^ | -3,67 | 7,994 | 1,94E-04 | 1,53E-04 | Duchess | 113381239 |  |
| Cer 34:2;O3 | Sphingolipids | + | 574,479889 | [M+Na]^+^ | -1,21 | 7,157 | 5,88E-06 | 6,93E-06 | Duchess | 113381280 |  |
| Cer 34:2;O3 | Sphingolipids | + | 574,480213 | [M+Na]^+^ | -0,62 | 7,733 | 7,28E-04 | 5,05E-04 | Duchess | 113381132 |  |
| Cer 34:1;O4 | Sphingolipids | + | 592,492249 | [M+Na]^+^ | 1,89 | 6,869 | 1,96E-13 | 5,21E-12 | Duchess | 113381239 |  |
| Cer 35:0;O3 | Sphingolipids | + | 608,502036 | [M+K]^+^ | 0,96 | 7,626 | 1,96E-03 | 1,23E-03 | Duchess | 113381312 |  |
| Cer 36:1;O3 | Sphingolipids | + | 626,509898 | [M+2Na-H] ^+^ | 0,69 | 6,221 | 4,96E-12 | 6,24E-11 | Duke | 113381196 |  |
| Cer 44:1;O | Sphingolipids | + | 684,662799 | [M+Na]^+^ | -0,19 | 8,692 | 3,36E-08 | 8,26E-08 | Duchess | 113381078 |  |
| Cer 44:0;O2 | Sphingolipids | + | 718,647379 | [M+K]^+^ | -0,07 | 8,680 | 4,70E-07 | 7,89E-07 | Duchess | 559264 | 0011771 |
| HexCer 34:1;O2 | Sphingolipids | + | 722,556255 | [M+Na]^+^ , [M+2Na-H]^+^ , [M+K]^+^ | 2,99 | 7,472 | 2,68E-10 | 1,57E-09 | Duchess | 113381871 |  |
| Cer 46:1;O2 | Sphingolipids | + | 728,691500 | [M+Na]^+^ | 3,39 | 8,523 | 5,11E-11 | 4,04E-10 | Duchess | 34448758 |  |
| Cer 45:0;O2 | Sphingolipids | + | 732,662692 | [M+H]^+^ | 3,29 | 8,716 | 1,41E-09 | 6,18E-09 | Duchess | 113381272 |  |
| Cer 48:1;O2 | Sphingolipids | + | 756,722222 | [M+Na]^+^ | 2,45 | 8,874 | 3,81E-11 | 3,18E-10 | Duchess | 57259533 |  |
| Cer 47:0;O2 | Sphingolipids | + | 760,693991 | [M+K]^+^ | -0,51 | 8,801 | 3,10E-08 | 7,69E-08 | Duchess | 113381275 |  |
| Cer 47:1;O3 | Sphingolipids | + | 774,671861 | [M-H_2_O+H]^+^ | -2,11 | 8,478 | 8,77E-06 | 9,79E-06 | Duchess | 113381162 |  |
| Cer 48:2;O2 | Sphingolipids | + | 776,685903 | [M+2Na-H]^+^ | -1,15 | 8,789 | 4,05E-07 | 6,90E-07 | Duchess | 113381223 |  |
| Cer 49:1;O4 | Sphingolipids | + | 802,723927 | [M+Na]^+^ | -2,55 | 9,463 | 1,44E-04 | 1,17E-04 | Duchess | 113381169 |  |
| Hex2Cer 39:0;O2 | Sphingolipids | + | 978,686502 | [M+2Na-H]^+^ | 3,93 | 0,522 | 6,05E-07 | 9,76E-07 | Duke | 113381841 |  |
| MIPC 42:0;O3 | Sphingolipids | - | 1052,701277 | [M-H_2_O-H]^-^ | -0,68 | 0,696 | 1,08E-09 | 4,85E-08 | Duke | 113381693 |  |
| LPE O-20:0 | Glycerophospholipids | + | 496,377630 | [M+H]^+^ | 3,00 | 5,768 | 1,24E-06 | 1,79E-06 | Duchess | 113376043 |  |
| PE-NMe(32:1) | Glycerophospholipids | + | 704,523849 | [M-H_2_O+H]^+^ | 1,91 | 8,442 | 6,23E-03 | 3,52E-03 | Duchess | 74849826 | 0112952 |
| PE(P-32:0) | Glycerophospholipids | + | 714,483669 | [M+K]^+^ | 0,37 | 4,935 | 3,65E-05 | 3,43E-05 | Duchess | 24768436 | 0008949 |
| PE(32:4) | Glycerophospholipids | + | 722,417523 | [M+K]^+^ | 2,62 | 6,719 | 2,64E-06 | 3,43E-06 | Duke | 113375428 |  |
| PE(P-33:2) | Glycerophospholipids | + | 724,467261 | [M+K]^+^ | -0,78 | 4,908 | 1,16E-04 | 9,63E-05 | Duke | 113375989 |  |
| PE 32:1 | Glycerophospholipids | + | 728,464210 | [M+K]^+^ | 2,14 | 7,356 | 6,32E-04 | 4,45E-04 | Duke | 24768333 | 0008828 |
| PE(40:9) | Glycerophospholipids | + | 768,493260 | [M-H_2_O+H]^+^ | -3,85 | 4,973 | 7,73E-04 | 5,32E-04 | Duke | 24768916 | 0009465 |
| PE(36:4) | Glycerophospholipids | + | 778,481484 | [M+K]^+^ | 4,23 | 7,783 | 3,72E-05 | 3,48E-05 | Duchess | 59698654 | 0009062 |
| PE-NMe2(38:8) | Glycerophospholipids | + | 826,480596 | [M+Na]^+^ , [M+K]^+^ | 2,84 | 8,109 | 2,61E-07 | 4,74E-07 | Duke | 74850957 | 0114169 |
| PE-NMe2(38:7) | Glycerophospholipids | + | 812,519512 | [M+H]^+^ | 3,34 | 5,030 | 2,43E-03 | 1,50E-03 | Duke | 74851094 | 0114307 |
| PE(24:6) | Glycerophospholipids | + | 1107,643723 | [2M+H]^+^ | 2,51 | 6,669 | 1,69E-11 | 1,66E-10 | Duke | 24769380 | 0011499 |
| LPE O-19:0;O | Glycerophospholipids | - | 542,344872 | [M+FA-H]- | -2,97 | 9,188 | 1,04E-03 | 4,42E-03 | Duke | 113376057 |  |
| PC(O-36:0) | Glycerophospholipids | + | 399,819887 | [M+H+Na]^2+^ | -2,88 | 0,602 | 3,97E-09 | 1,41E-08 | Duke | 24822862 | 0013406 |
| PC(14:0) | Glycerophospholipids | - | 466,292196 | [M-H]^-^ | -3,65 | 8,603 | 1,19E-09 | 4,98E-08 | Duke | 21403166 |  |
| PC(15:1) | Glycerophospholipids | - | 478,292777 | [M-H]^-^ | -2,35 | 8,431 | 2,80E-07 | 2,87E-06 | Duke | 113375408 |  |
| lysoPC(22:1) | Glycerophospholipids | + | 622,383051 | [M+2Na-H]^+^ | 1,94 | 4,335 | 2,39E-04 | 1,84E-04 | Duchess | 24766534 | 0010399 |
| PC(32:0) | Glycerophospholipids | + | 735,579944 | [M+H]^+^ | 4,37 | 7,541 | 1,99E-04 | 1,56E-04 | Duchess | 140912 |  |
| P-IsoPGF2-PC | Glycerophospholipids | + | 876,530083 | [M+2Na-H]^+^ | -4,38 | 7,775 | 1,85E-04 | 1,46E-04 | Duchess | 113386137 |  |
| PC(44:6) | Glycerophospholipids | + | 912,647062 | [M+Na]^+^ | 1,99 | 5,749 | 1,49E-13 | 4,29E-12 | Duchess | 24767211 | 0008551 |
| MIPC 40:0;O2 | Sphingolipids | + | 1066,652856 | [M+K]^+^ | -3,8 | 0,418 | 4,04E-07 | 6,89E-07 | Duke | 113381698 |  |
| PS(P-32:0) | Glycerophospholipids | - | 700,489639 | [M-H2O-H]^-^ | -3,69 | 8,778 | 1,52E-02 | 4,79E-02 | Duchess | 113376688 |  |
| PS(O-34:0) | Glycerophospholipids | + | 750,561585 | [M+H]^+^ | -3,68 | 7,222 | 1,98E-05 | 1,99E-05 | Duke | 113376638 |  |
| PHODA-PS (PS 28:2;O2) | Glycerophospholipids | + | 752,369922 | [M+2Na-H]^+^ | -3,09 | 7,445 | 5,54E-05 | 4,97E-05 | Duke | 113380879 |  |
| PS(31:3) | Glycerophospholipids | + | 760,415785 | [M+2Na-H]^+^ | 3,04 | 7,537 | 2,97E-03 | 1,79E-03 | Duke | 113376304 |  |
| PS(33:1) | Glycerophospholipids | + | 792,472596 | [M+2Na-H]^+^ | -4,78 | 7,868 | 3,96E-03 | 2,33E-03 | Duke | 113376323 |  |
| PS(O-36:4) | Glycerophospholipids | + | 808,491854 | [M+K]^+^ | 3,82 | 7,244 | 2,36E-04 | 1,82E-04 | Duchess | 113376597 |  |
| PS(46:4) | Glycerophospholipids | + | 962,623939 | [M+K]^+^ | -0,84 | 8,236 | 7,11E-05 | 6,21E-05 | Duchess | 74876256 | 112813 |
| PA(34:6) | Glycerophospholipids | + | 665,420952 | [M+H]^+^ , [M+Na]^+^ , [M+K]^+^ | 4,93 | 6,167 | 1,15E-08 | 3,34E-08 | Duchess | 74876570 | 0114803 |
| PA(32:4) | Glycerophospholipids | + | 679,371373 | [2M+Na]^+^ | 1,04 | 7,214 | 1,49E-03 | 9,64E-04 | Duke | 74876721 | 0114974 |
| PA(38:9) | Glycerophospholipids | + | 737,414965 | [M+Na]^+^ | -0,39 | 7,399 | 8,60E-05 | 7,38E-05 | Duke | 74876788 | 0115044 |
| SM(d36:1) | Sphingolipids | + | 731,608559 | [M+H]^+^ | 3,29 | 6,818 | 6,85E-12 | 8,07E-11 | Duke | 4956085 | 0001348 |
| SM(d42:1) | Sphingolipids | + | 837,680744 | [M+Na]^+^ | -1,50 | 8,813 | 1,38E-07 | 2,73E-07 | Duke | 24846875 |  |
| PIM1(37:3) | Glycerophospholipids | + | 530,301145 | [M+H+Na]^2+^ | -4,04 | 4,831 | 1,27E-05 | 1,35E-05 | Duchess | 113380500 |  |
| PI(35:3) | Glycerophospholipids | + | 847,532005 | [M+H]^+^ | -1,51 | 8,340 | 6,63E-06 | 7,72E-06 | Duke | 113378046 |  |
| PI(34:4) | Glycerophospholipids | + | 853,483364 | [M+Na]^+^ | -0,44 | 8,724 | 7,04E-05 | 6,15E-05 | Duke | 113378024 |  |
| PI(40:3) | Glycerophospholipids | - | 915,596313 | [M-H]^-^ , [M+FA-H]^-^ | -0,52 | 10,044 | 2,11E-03 | 8,36E-03 | Duke | 113378203 |  |
| Glc-GP(18:0/20:0) | Glycerophospholipids | + | 939,586754 | [M+2Na-H] ^+^ | -4,65 | 7,771 | 5,98E-06 | 7,04E-06 | Duchess | 113380437 |  |
| PI-Cer(d46:0) | Sphingolipids | - | 986,682424 | [M+K-2H]^-^ | -0,91 | 9,612 | 1,59E-08 | 2,68E-07 | Duke | 26332823 |  |
| LPIM4(19:2) | Glycerophospholipids | + | 1259,530227 | [M+H]^+^ , [M+Na]^+^ | -0,10 | 8,708 | 2,03E-04 | 1,59E-04 | Duke | 113380772 |  |
| LPIM4(18:2) | Glycerophospholipids | + | 1227,505022 | [M-H_2_O+H]^+^ | 0,69 | 9,059 | 5,31E-05 | 4,79E-05 | Duke | 113380769 |  |
| CL(32:0) | Glycerophospholipids | + | 538,293374 | [M+Na]^+^ , [M+2Na]^2+^ , [M+H+Na]^2+^ | -3,88 | 4,831 | 5,57E-09 | 1,85E-08 | Duchess | 74878491 | 0116812 |
| l-homoserine lactone | Lactones | + | 84,044844 | [M-H_2_O+H]^+^ | 4,51 | 0,629 | 1,08E-06 | 1,60E-06 | Duchess | 393441 |  |
| piperidine | Heteroaromatic compounds | + | 86,096830 | [M+H]^+^ , [M+2Na-H] ^+^ | 4,75 | 0,663 | 8,01E-09 | 2,47E-08 | Duke | 7791 | 34301 |
| uracil | Heteroaromatic compounds | + | 95,024526 | [M+H]^+^ , [M-H_2_O+H]^+^ | 4,81 | 0,609 | 1,35E-05 | 1,43E-05 | Duchess | 1141 | 0000300 |
| L-lysine | Amino acids | + | 129,102693 | [M-H_2_O+H]^+^ | 3,09 | 0,531 | 1,01E-08 | 2,99E-08 | Duchess | 5747 | 0000182 |
| Arginine | Amino acids | - | 211,060135 | [M+K-2H]^-^ | -0,86 | 3,094 | 1,20E-08 | 2,18E-07 | Duke | 6082 | 0000517 |
| cucurbitine | Amino acids | + | 113,071347 | [M-H_2_O+H]^+^ | 3,08 | 0,594 | 1,02E-06 | 1,52E-06 | Duke | 368099 |  |
| 1-indanone | Aryl alkyl ketones | + | 115,054692 | [M-H_2_O+H]^+^ | 3,56 | 8,672 | 1,24E-02 | 6,66E-03 | Duchess | 6479 | 0059602 |
| tyramine | Substituted aromatic compounds | + | 120,080966 | [M-H_2_O+H]^+^ | 1,41 | 2,908 | 6,53E-07 | 1,04E-06 | Duke | 5408 | 0000306 |
| phenethylamine | Substituted aromatic compounds | + | 122,096668 | [M+H]^+^ | 2,00 | 6,750 | 2,52E-03 | 1,55E-03 | Duchess | 13856352 | 0012275 |
| 4,5-diethyl-2-methoxyloazole | Heteroaromatic compounds | + | 122,096921 | [M-H_2_O+H]^+^ | 3,55 | 0,583 | 2,10E-08 | 5,52E-08 | Duchess | 30777209 | 0037870 |
| hydroxyproline | Amino acids | + | 132,065745 | [M+H]^+^ , [M-H_2_O+H]^+^ | 1,72 | 0,629 | 1,80E-05 | 1,83E-05 | Duchess | 802 | 0000725 |
| 3-[(5-methyl-2-furanyl]-1h-pyrrol | Heteroaromatic compounds | + | 144,080761 | [M-H_2_O+H]^+^ | -0,07 | 2,892 | 1,48E-06 | 2,09E-06 | Duchess | 30777403 | 0040042 |
| L-carnitine | Amino acids | + | 144,101433 | [M-H_2_O+H]^+^ | -2,94 | 0,645 | 9,79E-09 | 2,91E-08 | Duchess | 10455 | 0000062 |
| eugenol quinone methide | Substituted aromatic compounds | + | 145,064086 | [M-H_2_O+H]^+^ | -4,38 | 7,441 | 7,02E-06 | 8,11E-06 | Duchess | 30790798 |  |
| 3-hydroxy-3-methyloxindole | Heteroaromatic compounds | + | 146,060747 | [M-H_2_O+H]^+^ | 4,36 | 4,747 | 1,90E-10 | 1,19E-09 | Duchess | 133151 | 0004186 |
| halostachine | Substituted aromatic compounds | + | 152,106438 | [M+H]^+^ | -3,67 | 0,575 | 4,92E-07 | 8,21E-07 | Duchess | 889 | 0001387 |
| 2-phenylpyridine | Heteroaromatic compounds | + | 156,080073 | [M+H]^+^ | -4,55 | 2,689 | 4,22E-04 | 3,08E-04 | Duchess | 13286 |  |
| 4-phenylpyridine | Heteroaromatic compounds | + | 156,080533 | [M+H]^+^ | -1,38 | 3,340 | 9,31E-07 | 1,40E-06 | Duchess | 13062 | 0033123 |
| 5-butyl-4-methyl-2-propyloxazole | Heteroaromatic compounds | + | 164,142896 | [M-H_2_O+H]^+^ | -2,66 | 0,663 | 3,31E-07 | 5,80E-07 | Duchess | 30777213 | 0037897 |
| methionine | Amino acids | + | 172,039996 | [M+Na]^+^ | -1,88 | 4,411 | 2,86E-07 | 5,11E-07 | Duchess | 5907 | 00696 |
| loganin | Terpenoid compounds | + | 207,074657 | [M+Na+H]^2+^ | 0,59 | 0,658 | 2,65E-05 | 2,58E-05 | Duchess | 79111 |  |
| beta-estradiol | Steroids and steroid derivatives | + | 255,174864 | [M-H_2_O+H]^+^ | 1,87 | 8,878 | 1,80E-09 | 7,48E-09 | Duchess | 5554 | 0000151 |
| n-lactoyl-tryptophan | Amino acids | + | 259,107583 | [M-H_2_O+H]^+^ | -0,44 | 4,946 | 1,44E-09 | 6,26E-09 | Duchess | 59664334 | 0062178 |
| arginyl-hydroxyproline | Amino acids | + | 270,155143 | [M-H_2_O+H]^+^ | -3,24 | 0,539 | 4,08E-08 | 9,69E-08 | Duchess | 35032787 | 0028710 |
| capsiamide | Acetamides | + | 270,279713 | [M+H]^+^ , [M+Na]^+^ | 2,17 | 9,619 | 1,76E-04 | 1,40E-04 | Duchess | 43094 | 0040940 |
| benzyl cinnamate | Substituted aromatic compounds | + | 283,069663 | [M+2Na-H]^+^ | -3,70 | 2,762 | 5,84E-07 | 9,46E-07 | Duchess | 21391699 | 0040286 |
| octanoylglucuroniden | Carbohydrates | + | 359,109445 | [M+K]^+^ | -2,62 | 5,164 | 3,08E-06 | 3,93E-06 | Duke | 113087 | 0010347 |
| dihydroxycitracridone I | Heteroaromatic compounds | + | 388,139412 | [M+H]^+^ | 0,85 | 0,626 | 1,97E-05 | 1,99E-05 | Duchess | 35013367 | 0031401 |
| amphibine H | Amino acids | + | 606,327975 | [M+H]^+^ | -1,08 | 3,845 | 2,77E-08 | 6,98E-08 | Duchess | 35032851 | 0029333 |
| imidazoleacetic acid riboside | Heteroaromatic compounds | - | 239,066454 | [M-H_2_O-H]^-^ | -3,41 | 10,563 | 7,29E-05 | 3,95E-04 | Duchess | 389469 | 0002331 |
| 5'-phosphoribosyl-N-formylglycinamidine | Carbohydrates | - | 306,011147 | [M+Na-H]^-^ | 1,00 | 4,646 | 8,41E-03 | 2,85E-02 | Duchess | 17216355 | 0006211 |
| catechin 5-O-beta-D-glucopyranoside-4'-Me | Flavonoids | - | 511,144917 | [M+FA-H]^-^ | -1,73 | 4,906 | 1,07E-02 | 3,50E-02 | Duke | 24842566 |  |
| naringin chalcone | Flavonoids | - | 579,169689 | [M+FA-H]^-^ | -3,85 | 4,321 | 5,92E-04 | 2,65E-03 | Duke | 30791938 |  |
| narirutin | Flavonoids | - | 579,169782 | [M-H]^-^ | -3,69 | 4,240 | 2,05E-12 | 1,86E-09 | Duke | 390871 |  |
| neohesperidin | Flavonoids | - | 609,181177 | [M-H]^-^ | -2,14 | 4,420 | 2,39E-10 | 2,33E-08 | Duke | 390879 |  |
| hesperidin | Flavonoids | - | 609,181565 | [M-H]^-^ | -1,60 | 4,481 | 5,95E-10 | 3,66E-08 | Duke | 10176 |  |
| 5-(4'-hydroxyphenyl)-gamma-valerolactone-4'-O-glucuronide | Lactones | - | 385,163625 | [M+Na-2H]- | 0,98 | 10,613 | 2,07E-06 | 1,62E-05 | Duke | 35031677 | 0059992 |
| nemanilactone A | Lactones | + | 137,060337 | [M-H_2_O+H]^+^ | 4,14 | 4,885 | 7,77E-07 | 1,21E-06 | Duke | 78436064 |  |
| 4-hydroxy-3-methyl-6-[(1E,3E)-1,3-pentadien-1-yl]-2H-pyran-2-one | Lactones | + | 175,074865 | [M-H_2_O+H]^+^ | -2,56 | 5,825 | 1,30E-04 | 1,07E-04 | Duchess | 78434945 |  |
| (2E)-3-{4-[4-Hydroxy-4-(hydroxymethyl)cyclohexyl]-2-furyl}-2-methylacrylamide | Heteroaromatic compounds | + | 262,144317 | [M-H_2_O+H]^+^ | 1,94 | 7,449 | 3,33E-10 | 1,88E-09 | Duchess | 78434648 |  |
| acremeremophilane H | Terpenoid compounds | + | 277,180288 | [M+H]^+^ , [M-H_2_O+H]^+^ | 1,68 | 7,437 | 9,71E-12 | 1,07E-10 | Duchess | 58196724 |  |
| 2,4,6,8-tetramethyl-3,4-dihydroxydec-8(9)-enolide | Lactones | + | 279,157776 | [M+Na]^+^ | 4,32 | 6,949 | 2,97E-03 | 1,79E-03 | Duchess | 78441936 |  |
| nannochelin C | Substituted aromatic compounds | + | 371,152151 | [M+2H]^2+^ | -0,97 | 0,663 | 7,49E-03 | 4,18E-03 | Duke | 20144000 |  |
| reveromycin M | Antibiotic compounds (Heteroaromatic compounds) | + | 386,169554 | [M+2Na]^2+^ | -1,16 | 0,668 | 4,51E-04 | 3,27E-04 | Duke | 78439672 |  |
| isomigrastatin | Lactones | + | 512,263323 | [M+Na]^+^ | 2,98 | 7,241 | 9,88E-05 | 8,37E-05 | Duchess | 13083250 |  |
| lavendomycin | Antibiotic compounds | + | 667,389150 | [M+H]^+^ | 0,88 | 7,982 | 3,37E-06 | 4,25E-06 | Duchess | 78443026 |  |
| versixanthone E/F | Substituted aromatic compounds | + | 671,194599 | [M+H]^+^ | -3,63 | 5,187 | 4,47E-10 | 2,39E-09 | Duke | 78436425 |  |
| bacteriohop-6-enetetrol carbapesudopentose ether | Terpenoid compounds | + | 706,524584 | [M+H]^+^ , [M+Na]^+^ | -0,90 | 7,098 | 2,59E-07 | 4,72E-07 | Duchess | 78445146 |  |
| bacteriohopanetetrol carbapesudopentose ether | Terpenoid compounds | + | 708,540660 | [M+H]^+^ | -0,35 | 7,248 | 3,16E-11 | 2,73E-10 | Duchess | 78445339 |  |
| 21-hydroxyoligomycin A | Antibiotic compounds | + | 789,513228 | [M-H_2_O+H]^+^ | 3,15 | 7,453 | 6,72E-05 | 5,90E-05 | Duke | 17272226 |  |
| rifamycin Y | Antibiotic compounds | + | 792,285500 | [M+Na]^+^ | 2,24 | 0,658 | 2,41E-05 | 2,37E-05 | Duke | 57522872 |  |
| SF-837 A4 | Antibiotic compounds | + | 808,446961 | [M+Na]^+^ | 1,99 | 8,002 | 4,15E-06 | 5,09E-06 | Duke | 78444929 |  |
| kujimycin A | Antibiotic compounds | + | 813,460545 | [M+Na]^+^ | -0,22 | 7,606 | 3,97E-06 | 4,90E-06 | Duke | 78443185 |  |
| oligomycin C | Lactones | + | 819,502330 | [M+2Na-H]^+^ | 3,83 | 7,694 | 1,25E-02 | 6,74E-03 | Duke | 16735942 |  |
| Shengjimycin E | Antibiotic compounds | + | 909,571417 | [M-H_2_O+H]^+^ | 3,42 | 7,691 | 1,18E-05 | 1,27E-05 | Duchess | 78445013 |  |
| GGL.3 | Glycerolipids | + | 951,565729 | [M+2Na-H]^+^ | 3,25 | 8,478 | 1,51E-06 | 2,12E-06 | Duchess | 78444110 |  |
| atacamycin A | Lactones | + | 1001,630029 | [2M+H]^+^ | -4,81 | 8,344 | 3,81E-03 | 2,25E-03 | Duchess | 78444650 |  |
| saponaceolide J | Lactones | + | 1001,631624 | [2M+H]^+^ | -3,21 | 8,125 | 1,31E-03 | 8,58E-04 | Duchess | 78441012 |  |
| RS-22A | Antibiotic compounds | + | 1036,628213 | [M-H_2_O+H]^+^ | -3,21 | 8,559 | 1,88E-05 | 1,90E-05 | Duchess | 78444635 |  |
| TMC-34 | Lactones | + | 1038,647563 | [M-H_2_O+H]^+^ | 0,32 | 8,466 | 4,39E-05 | 4,04E-05 | Duchess | 78444305 |  |
| Neocopiamycin A | Antibiotic compounds | + | 1066,639154 | [M+H]^+^ , [M+Na]^+^ , [M+K]^+^ , [M+2Na-H]^+^ | -0,56 | 8,623 | 6,13E-09 | 2,00E-08 | Duchess | 21247624 |  |
| Thermolide | Lactones | + | 1113,699064 | [2M+Na]^+^ | -2,68 | 8,121 | 5,52E-13 | 1,14E-11 | Duke | 78440766 |  |
| curvulalide | Lactones | - | 235,059691 | [M+Na-2H]^-^ | 4,21 | 4,194 | 1,08E-03 | 4,59E-03 | Duke | 27022359 |  |
| Penipacid B | Substituted aromatic compounds | - | 263,139298 | [M-H]^-^ | -3,13 | 2,585 | 1,19E-02 | 3,86E-02 | Duke | 30771322 |  |
| vegfrecine | Substituted aromatic compounds | - | 310,023350 | [M+K-2H]^-^ | -0,75 | 0,669 | 1,16E-07 | 1,35E-06 | Duke | 78435049 |  |
| Asperversin G | Terpenoid compounds | - | 381,172708 | [M-H_2_O-H]^-^ | 4,87 | 10,578 | 3,78E-03 | 1,41E-02 | Duchess | 71049007 |  |
| pseudoargiopinin III | Substituted aromatic compounds | - | 410,161411 | [M+FA-H]^-^ | -2,13 | 9,624 | 1,01E-02 | 3,33E-02 | Duchess | 24720482 |  |
| 5-O-alfa-L-rhamosyltylactone | Lactones | - | 521,309436 | [M-H_2_O-H]^-^ | -4,73 | 9,065 | 3,94E-04 | 1,83E-03 | Duke | 78440368 |  |
| Gilvocarcin HE | Glycosides | - | 557,166030 | [M+FA-H]^-^ | -0,87 | 4,363 | 2,98E-08 | 4,48E-07 | Duke | 78438048 |  |
| Wollamide A | Antibiotic compounds | - | 790,404312 | [M+K-2H]^-^ | 2,62 | 8,618 | 6,79E-03 | 2,34E-02 | Duke | 78440470 |  |
| kanglemycin A | Antibiotic compounds | - | 1026,399522 | [M+FA-H]^-^ | 1,94 | 8,239 | 6,42E-09 | 1,40E-07 | Duke | 78442984 |  |
